# Supplementary material for: Contribution of epigenetic variation to adaptation in Arabidopsis
Source: Nat Commun. 2018 Oct 25;9:4446. doi: 10.1038/s41467-018-06932-5 (PMC6202389; doi:10.1038/s41467-018-06932-5)
Supplement: Supplementary file 1 — Supplementary Information [file 41467_2018_6932_MOESM1_ESM.pdf]

Epigenetic variation contributes to adaptation in *Arabidopsis*

Schmid and Heichinger *et al.*

Supplemental Information

Supplementary Figures

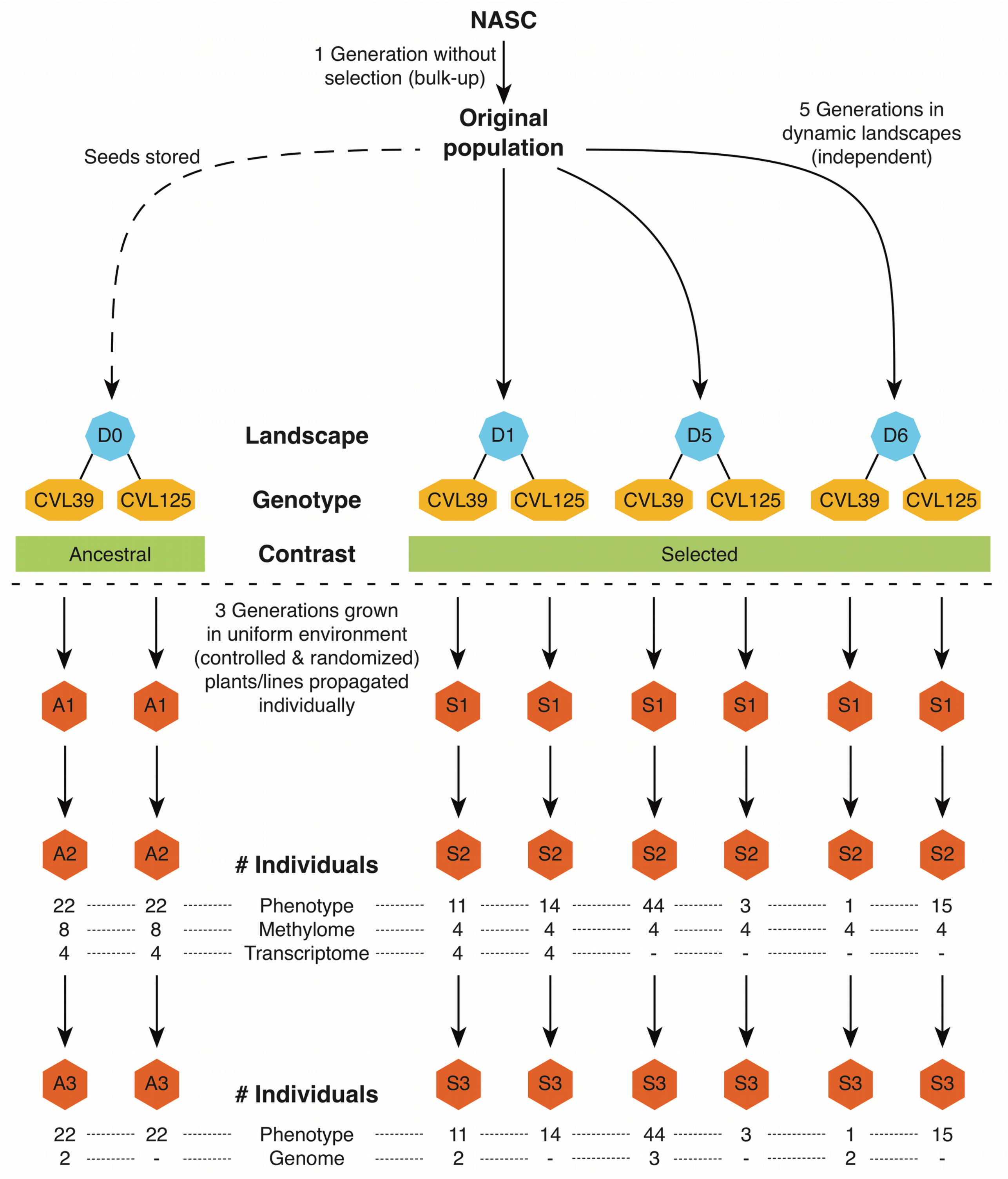

Supplementary Figure 1. Diagram of the propagation history of the lines analyzed in the different experiments. The original population consisted of 19 equally represented genotypes and was grown for five generations in a selective environment[1]. Two genotypes (CVL39 and CVL125) dominated the selected populations and were used for the present study. Offspring from the original population (D0) and the selected populations (originating from three independent, replicate selection experiments, i.e., landscapes D1, D5, and D6) were grown for three generations in a non-selective environment (controlled conditions and randomized plant locations). Phenotypes were measured in the second and third generation. Methylome and transcriptome were profiled in the second generation. Genomes were re-sequenced in the third generation.

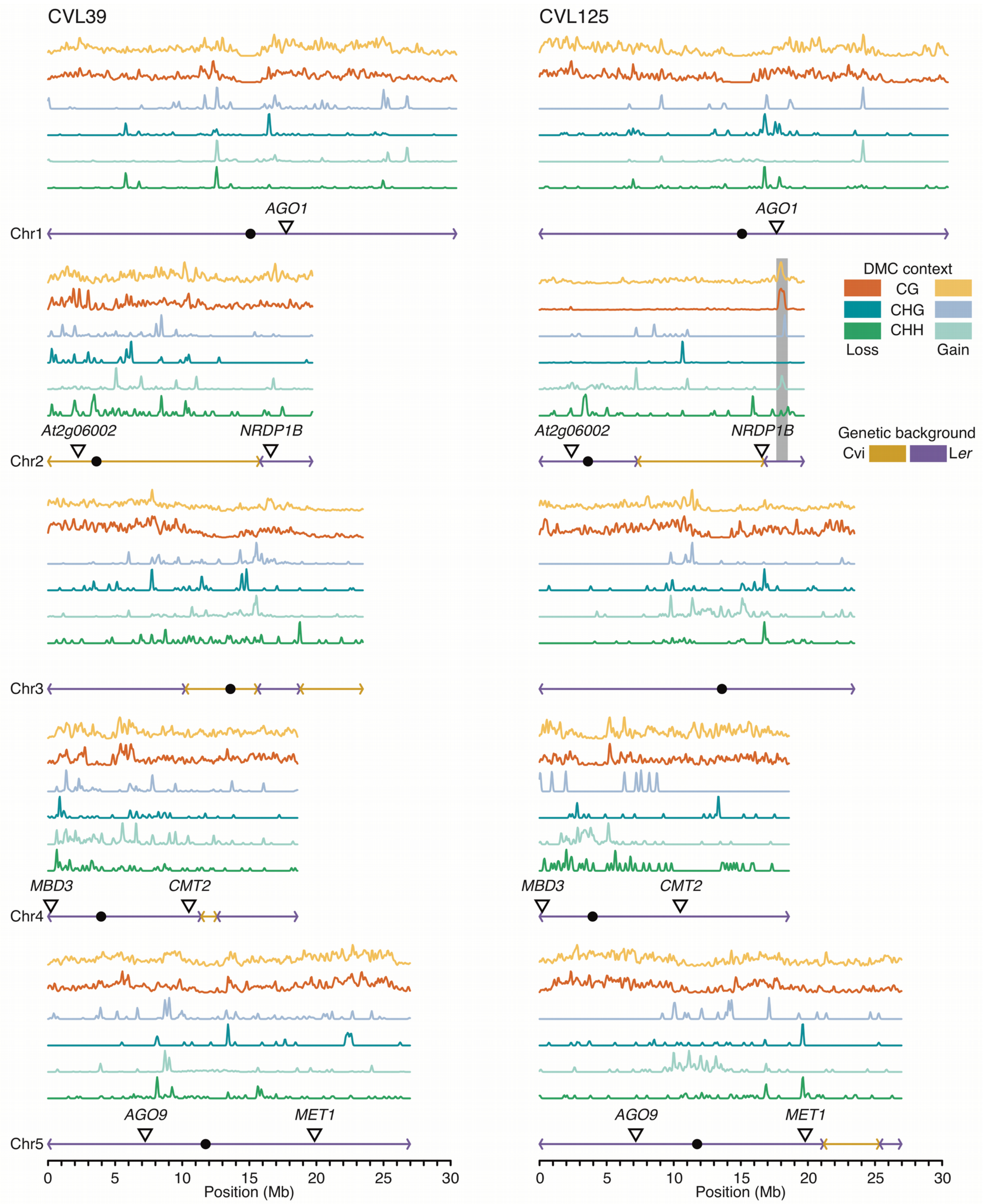

Supplementary Figure 2. Distribution of DMCs along the five *Arabidopsis* chromosomes. The parental origin of chromosome segments is demarcated (genetic background). Large filled circles indicate centromere positions. For CVL125, the DMC density on chromosome 2 is dominated by an enrichment in a region heterozygous for L*er*/Cvi (shaded in grey, see Methods, "Reference genomes for the recombinant inbred lines (RILs)"). The location of six major *trans*-acting loci described in [2] (*AGO1*, *NRPD1B*, *MBD3*, *CMT2*, *AGO9*, and *MET1*) and the epiallele *At2g06002* are shown as triangles.

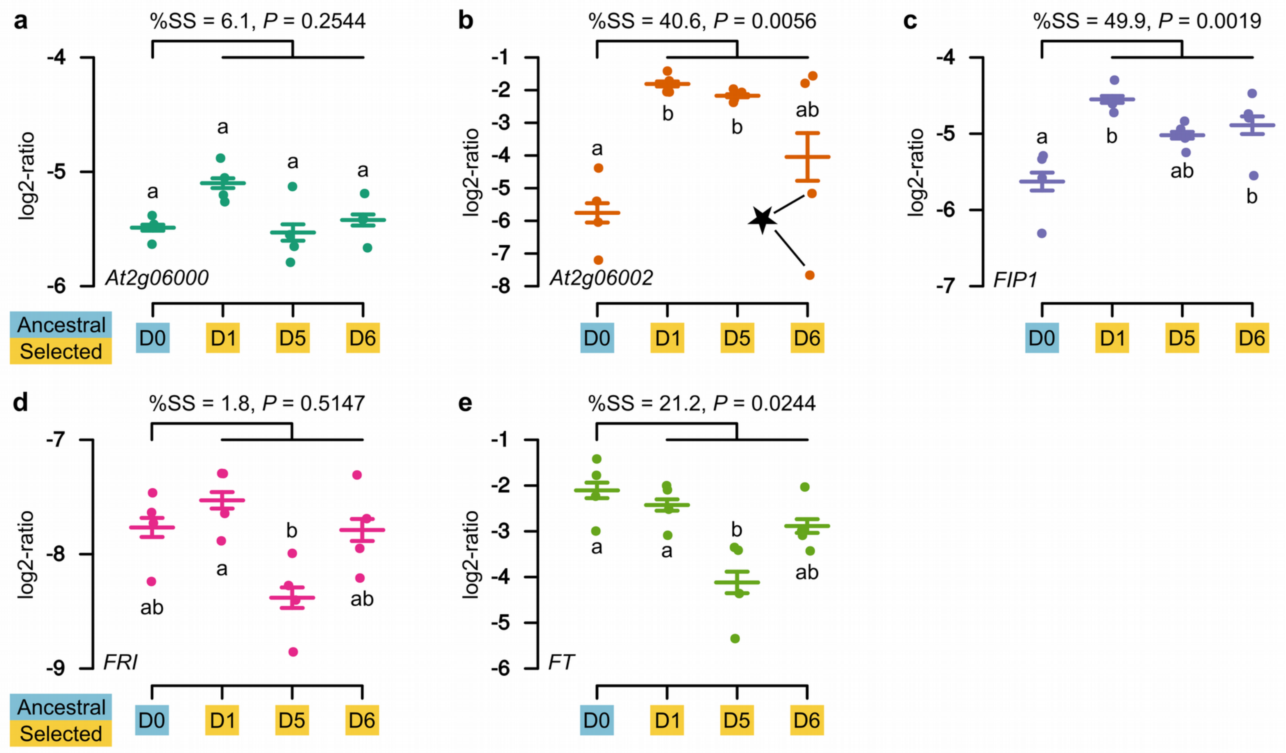

Supplementary Figure 3. Expression pattern of selected genes in 16 individuals of CVL125 for which DNA methylation data was available. Expression patterns were assessed by droplet digital PCR (ddPCR) with two reference genes (*PP2A* and *UBC9*). The y-axis corresponds to the log-ratio between the test and the geometric mean of the reference genes (log2(test gene count + 1) - log2(geometric mean reference gene counts + 1)). Long horizontal lines indicate the mean, short horizontal lines delimit plus/minus one standard error of the mean. Different letters indicate conditions that are significantly different from each other (two-sided *t*-test, adjusted for multiple testing, FDR < 0.05). Conditions with the same letter are not significantly different from each other. %SS and *P* on top of each panel correspond to the percentage of sum of squares explained by the contrast comparing the selected populations with the ancestral population and the corresponding *P*-value. a) *At2g06000*, b) *At2g06002*, c) *FIP1*, d) *FRI*, and e) *FT*. b) The asterisk marks the two individuals with high DNA methylation levels at *At2g06002* (Fig. 3b), which, in turn, have expression levels similar to the individuals of the ancestral population.

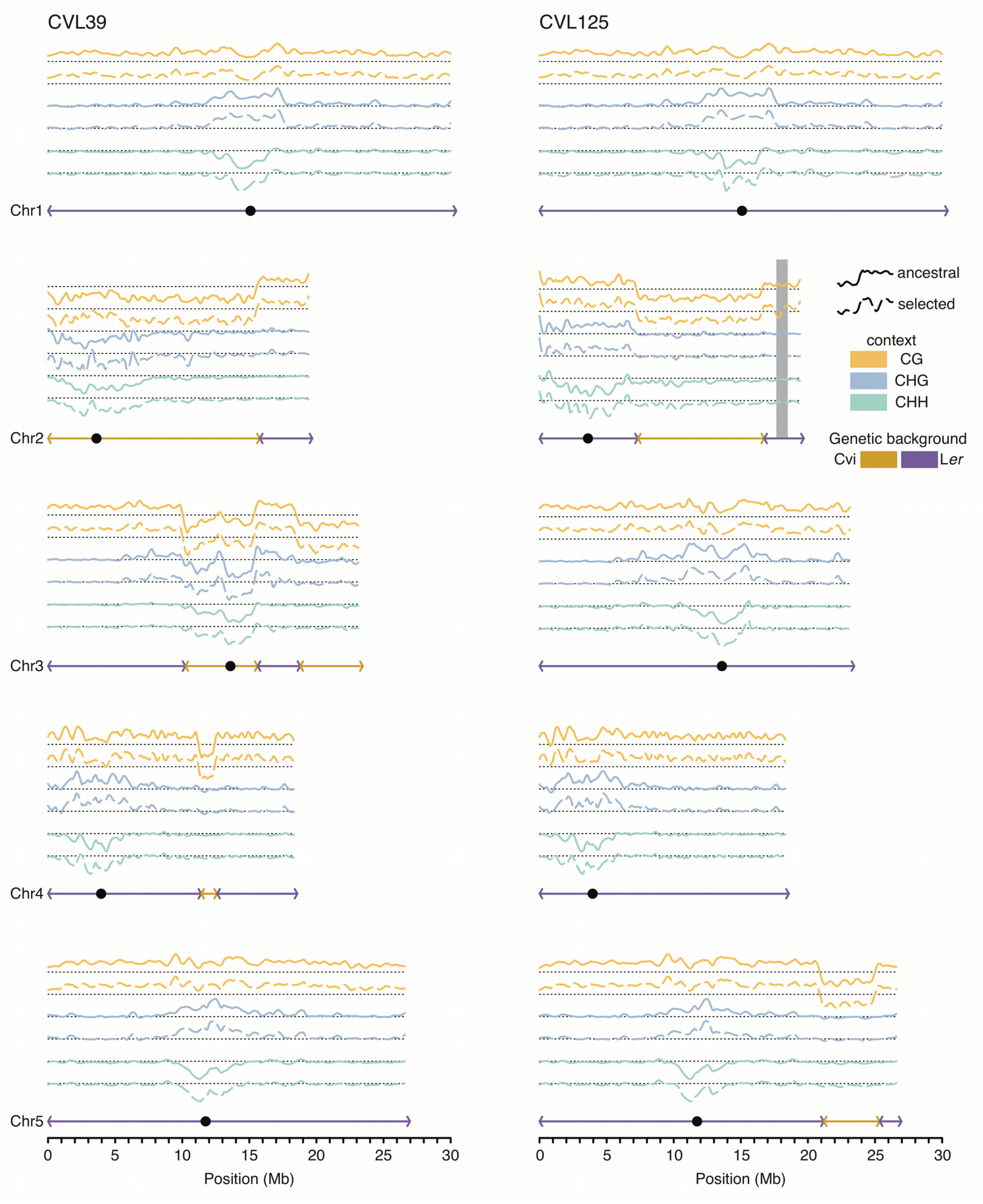

Supplementary Figure 4. Differences of average pairwise distances of ancestral and selected individuals to Cvi and L*er*. The average distance was calculated as the average distance to L*er* minus the average distance to Cvi; 10 kb bins). Solid and dashed lines for ancestral and selected individuals, respectively. Values above or below the dotted lines indicate higher similarity to L*er* or Cvi, respectively. The parental origin of chromosome segments is demarcated (genetic background). Large filled circles indicate centromere positions. The region heterozygous for L*er*/Cvi in CVL125 is shaded in grey (see Methods, "Reference genomes for the recombinant inbred lines (RILs)").

Supplementary Tables

Supplementary Table 1. List and description of SNPs identified by re-sequencing of two/seven individuals of the ancestral/selected populations of CVL39 (third generation).

chromosome	position	base call in individuals of the ancestral population		base call in selected populations	allele present in at least one individual of the ancestral population (incl. Sanger Seq.)	genetic context to which the SNP maps to; if intergenic, the gene up- and downstream of the SNP	amino acid change caused by the SNP	differentially expressed in CVL39
Chr1	19702600	G	G	A	N	AT1G52900, exon	Ser/Leu	N
Chr1	26665796	C	C	A	Y	intergenic, AT1G70710, AT1G70720	-	N
Chr1	27486029	G	G	A	Y	AT1G73080, exon	His/Arg	N
Chr3	3870189	A	A	G	N	AT3G12140, exon	Asn/Asn	N
Chr3	5955028	C	C	T	Y	AT3G17400, exon	Leu/Phe	N
Chr1	30196443	A	T	A	Y	AT1G80310, 3' UTR	-	N
Chr4	2908207	A	G	A	Y	AT4G05590, intron	-	N
Chr1	16733748	C	T/C	C	Y	AT1G44045 (TEG)	Ala/Ser	N
Chr2	8575225	T/C	C	C	Y	AT2TE35880 (TE)	-	-
Chr2	1108089	T/C	C	C	Y	intergenic, AT2G03640, AT2TE05000 (TE)	-	N
Chr2	11380307	A	A/G	A	Y	AT2TE49230 (TE)	-	-
Chr2	11643873	T/G	T/G	T	Y	AT2TE50570 (TE)	-	-
Chr5	9359805	T	T/C	C	Y	intergenic, AT5G26617, AT5G26622	-	N
Chr2	3759503	G	G	G or A/G	Y	AT2TE16230 (TE), AT2TE16235 (TE), AT2G09920 (TEG)	-	N

Supplementary Table 2. List of primer sequences used for sequencing of CVL39 SNPs (see Supplementary Table 1) and the ddPCR (see Supplementary Data 12).

SNP	Forward	Reverse
SNP1	AGGGCCCATCTAAACCTTTG	AACCACAGAGGATCCGACAC
SNP2	ATCATGCAGTGCGAAACAAC	AGGATTCTGAATGACCGAAA
SNP3	GCCATGGAAGGAAGTTGAGA	AGCCAACATCAAAACGCTCT
SNP4	GCCGACCCTGTGAGAATAAA	CAATTGGTGCGACTGGAAAT
SNP5	AAGCAGCAACGAAGGAGAAA	GAGCCTAGCGCAGTCTTTTG

Gene	Forward	Reverse	Reference
At2g06000	AAACGTCACTACATCCGGGAA	TCCTCGTTGATGGTTATGCG	
At2g06002	GAAGCTGTCATGGAGGATCGA	CATTAGCAGCAAGGACACGTG	
FIP1	GCTACGGCTTGCTCGTTCTTT	TCTCTCAAGATTCGCTGCCC	
FRI	TGCCTGATCGTGGTAAAGGGAAG	AGCACCGGCAATCTCATTCGAAC	3
FT	CTAGCAACCCTCACCTCCGA	GCAATGAGATTGTGTGTTACGA	4
PP2A	TAACGTGGCCAAAATGATGC	GTTCTCCACAACCGCTTGGT	5
UBC9	TCACAATTTCCAAGGTGCTGC	TCATCTGGGTTTGGATCCGT	

Supplementary References

1. Fakheran, S. *et al.* Adaptation and extinction in experimentally fragmented landscapes. *Proc Natl Acad Sci U S A* **107**, 19120-19125 (2010).

2. Kawakatsu, T. *et al.* Epigenomic diversity in a global collection of *Arabidopsis thaliana* accessions. *Cell* **166**, 492-505 (2016).

3. Schmalenbach, I., Zhang, L., Ryngajllo, M. & Jiménez-Gómez, J.M. Functional analysis of the Landsberg *erecta* allele of *FRIGIDA. BMC Plant Biol* **14:** 218 (2014).

4. Zhang *et al.* The Cyclophilin CYP20-2 Modulates the Conformation of BRASSINAZOLE-RESISTANT1, Which Binds the Promoter of *FLOWERING LOCUS D* to Regulate Flowering in *Arabidopsis*. *Plant Cell* **25**: 2504-2521 (2013).

5. Czechowski, T., Stitt, M., Altmann, T., Udvardi, M.K. & Scheible, W.-R. Genome-Wide Identification and Testing of Superior Reference Genes for Transcript Normalization in Arabidopsis . *Plant Physiology* **139**: 5-17 (2005).
