# Supplementary material for: Contribution of epigenetic variation to adaptation in Arabidopsis
Source: Nat Commun. 2018 Oct 25;9:4446. doi: 10.1038/s41467-018-06932-5 (PMC6202389; doi:10.1038/s41467-018-06932-5)
Supplement: Supplementary file 17 — Description of Additional Supplementary Files [file 41467_2018_6932_MOESM17_ESM.docx]

**Title:** Supplementary Data 1:
**Description:** ANOVA results for the phenotypes measures at the second and third generation. Averages and standard deviations for each phenotype given a population of origin and generation are given on the right.

**Title:** Supplementary Data 2:
**Description:** Alignment statistics of the Illumina bisulfite sequencing data.

**Title:** Supplementary Data 3:
**Description:** DMCs in CVL39.

**Title:** Supplementary Data 4:
**Description:** DMCs in CVL125.

**Title:** Supplementary Data 5:
**Description:** DMRs in CVL39 with at least five DMCs and a maximal distance between two neighboring DMCs of 50 bp.

**Title:** Supplementary Data 6:
**Description:** DMRs in CVL125 with at least five DMCs and a maximal distance between two neighboring DMCs of 50 bp.

**Title:** Supplementary Data 7:
**Description:** Number of DMCs and average methylation differences per gene in CVL39.

**Title:** Supplementary Data 8:
**Description:** Number of DMCs and average methylation differences per gene in CVL125.

**Title:** Supplementary Data 9:
**Description:** List of GO terms found to be significantly enriched in at least 50 out of 121 parameter combinations (minimal number of DMCs and minimal absolute change in methylation) in CVL39 and/or CVL125 (out of 372/415 terms tested in CVL39/CVL125).

**Title:** Supplementary Data 10:
**Description:** Genes with the gene ontology term "vegetative to reproductive phase transition of meristem" (GO:0010228) mapped by at least one DMC in any of the RILs.

**Title:** Supplementary Data 11:
**Description:** Gene expression values in individuals of the ancestral and selected populations (second generation).

**Title:** Supplementary Data 12:
**Description:** Results from the Droplet Digital PCR (ddPCR) assay. Expression values of test genes and reference genes from the reaction using the cDNA libraries (RT+, first table) or a mock library in which there was no cDNA (RT-, second table).

**Title:** Supplementary Data 13:
**Description:** Data underlying Figure 3e.

**Title:** Supplementary Data 14:
**Description:** Publicly available DNA methylation, expression, and phenotype data. DNA methylation and expression data from Schmitz et. al (2013) Nature 594: 193-8. Phenotype data from Atwell et al. (2010) Nature 465: 627-31.
